# Supplementary material for: Interfering With Contextual Fear Memories by Post-reactivation Administration of Propranolol in Mice: A Series of Null Findings
Source: Front Behav Neurosci. 2022 Jun 27;16:893572. doi: 10.3389/fnbeh.2022.893572 (PMC9272000; doi:10.3389/fnbeh.2022.893572)
Supplement: Supplementary file 1 [file Data_Sheet_1.docx]

**Interfering with contextual fear memories by post-reactivation administration of propranolol in mice: a series of null findings**

**Wouter R. Cox^1^, Leonidas Faliagkas^2^, Amber Besseling^2^, Rolinka van der Loo^2^, Sabine Spijker^2^, Merel Kindt^1^, Priyanka Rao-Ruiz^2*^**

^1^Department of Psychology, Clinical Psychology, University of Amsterdam, Amsterdam, The Netherlands

^2^Department of Molecular and Cellular Neurobiology, Center for Neurogenomics and Cognitive Research, Amsterdam Neuroscience, Vrije Universiteit Amsterdam, Amsterdam, The Netherlands

***Correspondence:**Priyanka Rao-Ruiz
p.rao@vu.nl

Supplementary Material

1. **Supplementary Results**

To rule out that the null findings in the present study could be explained by insufficient statistical power within the individual experiments, we performed additional analyses with the data of multiple experiments collapsed. All experiments were sorted by research instrument (TSE systems, Med Associates system), and drug (saline, propranolol, anisomycin). As Experiment 5 involved a different reactivation duration than all other experiments and Experiment 7 did not involve a retention test, these two experiments were excluded from this analysis. Across the experiments performed in a TSE system (Experiments 1–2), there was no effect of post-reactivation administration of propranolol versus saline on changes in freezing from memory reactivation to retention test (Drug × Session, *F*_1,70_ = 0.187, *p* = 0.667). For the collapsed data of experiments that were performed in a Med Associates system (Experiment 3–4, 6, 8, 9), this same analysis also showed no significant effect (*F*_1,129_ = 1.732, *p* = 0.190). Finally, when the data of all anisomycin conditions were collapsed (Experiment 6, 8, 9), no effect was found either (*F*_1,50_ = 0.394, *p* = 0.533). Therefore, low statistical power does not seem to be an explanation of the null findings in the present series of experiments.

To gain more insight into whether these null results are explained by low freezing during reactivation, we repeated these analyses while excluding animals that froze less than 10% of the total reactivation time. No significant effect of propranolol in a TSE system (F1,69 = 0.217, p = 0.643) was found, nor an effect of propranolol (F1,94 = 3.757, p = 0.056) or anisomycin (F1,63 = 0.157, p = 0.693) in a Med Associates system. Also, when we excluded experiments 4 and 9b (i.e., which showed particularly low levels of freezing), no amnestic effect of propranolol in a Med Associates system was found (F1,78 = 1.743, p = 0.191).

**
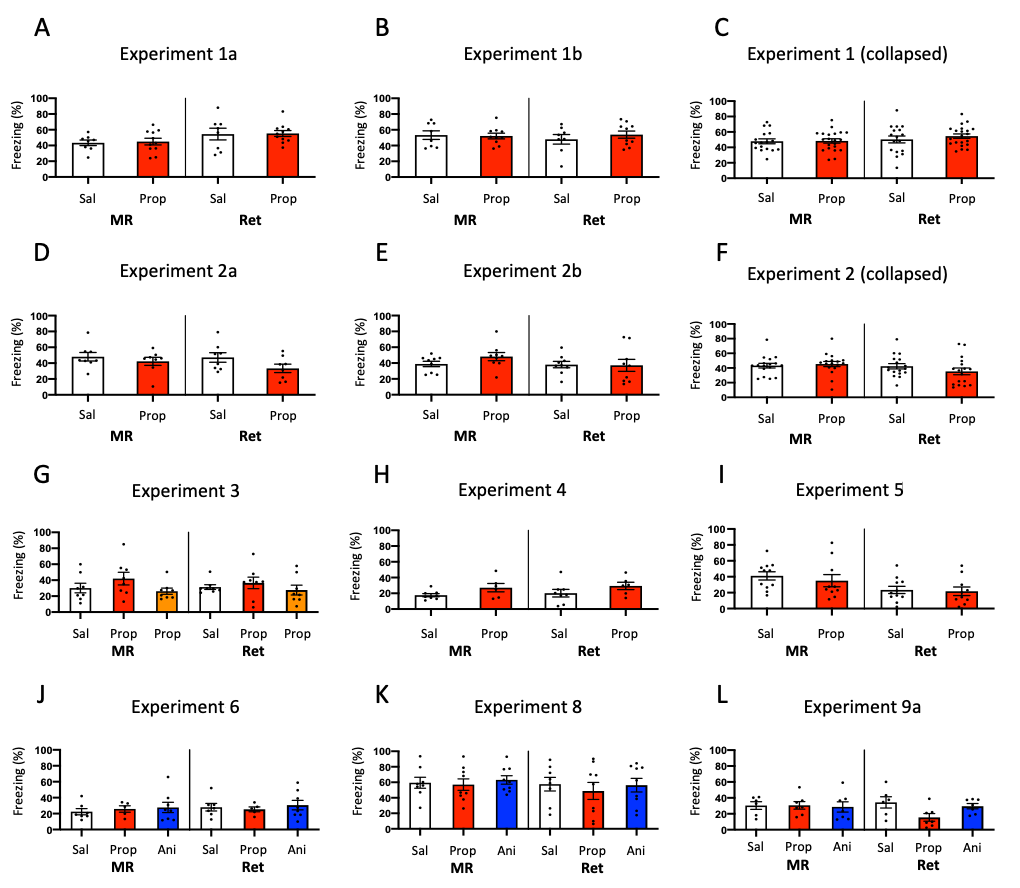

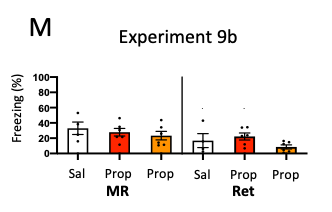

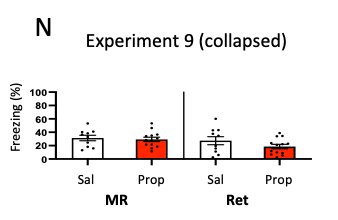
Figure. S1.** Analyses of Experiment 1–6, 8, and 9 in which animals that showed lower than 10% freezing during memory reactivation were excluded. Cond = conditioning, MR = memory reactivation, Ret = retention, Sal = saline, Prop = propranolol, Ani = anisomycin. (**A**) Experiment 1a. One mouse was excluded due to low freezing (propranolol condition). No Drug x Session interaction was found, *F*_1,17_ = 0.008, *p* = 0.929. (**B**) Experiment 1b. No mice showed freezing levels lower than 10% during memory reactivation. (**C**) Collapsed data of Experiment 1a and 1b. No Drug x Session interaction was found, *F*_1,35_ = 0.270, *p* = 0.607. (**D**) Experiment 2a. No mice showed freezing levels lower than 10% during memory reactivation. (**E**) Experiment 2b. No mice showed freezing levels lower than 10% during memory reactivation. (**F**) Collapsed data of Experiment 2a and 2b. (**G**) Experiment 3. Three mice were excluded (saline condition, *n* =1; propranolol condition, *n* = 1, propranolol double volume condition, *n* = 1). No Drug x Session interaction was found, *F*_2,21_ = 0.437, *p* = 0.652. (**H**) Experiment 4. Ten mice were excluded due to low freezing (saline condition, *n* = 4; propranolol condition, *n* = 6). No Drug x Session interaction was found, *F*_1,12_ = 0.002, *p* = 0.963. (**I**) Experiment 5. One mouse was excluded (propranolol condition). No Drug x Session interaction was found, *F*_1,19_ = 0.036, *p* = 0.852. (**J**) Experiment 6. Five mice were excluded (saline condition, *n* =1; propranolol condition, *n* = 4). No Drug x Session interaction was found, *F*_2,17_ = 0.422, *p* = 0.662. (**K**) Experiment 8. One mouse was excluded (saline condition). No Drug x Session interaction was found, *F*_2,23_ = 0.207, *p* = 0.815. (**L**) Experiment 9a. Seven mice were excluded (saline condition, *n* = 3; propranolol condition, *n* = 2; anisomycin condition, *n* = 2). A Drug x Session interaction was observed at trend level, *F*_2,17_ = 3.214, *p* = 0.065. (**M**) Experiment 9b. Eleven mice were excluded (saline condition, *n* = 5; propranolol condition, *n* = 3; propranolol double volume, *n* = 3). No Drug x Session interaction was found, *F*_2,13_ = 1.632, *p* = 0.233. (**N**) Collapsed data of Experiment 9a and 9b. No Drug x Session interaction was found, *F*_1,21_ = 1.151, *p* = 0.295. Error bars represent SEM. Filled circles indicate individual animals

**
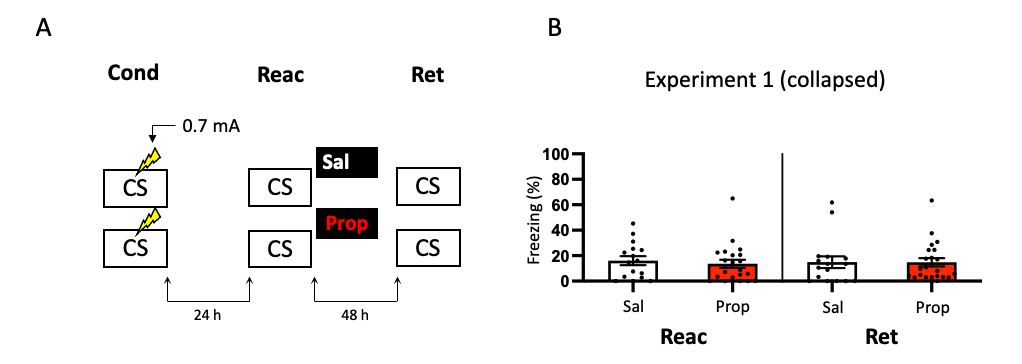
Fig. S2**. Results of Experiment 1 with freezing measured using a time threshold of 5 s, as in a previous study that used the same protocol (Rao-Ruiz et al., 2011). Cond = conditioning, MR = memory reactivation, Ret = retention, CS = conditioned stimulus (context), Sal = saline, Prop = propranolol. Error bars represent SEM. Filled circles indicate individual animals. Again, no effect of post-reactivation administration of propranolol was found on contextual fear (*F*_1,36_ = 0.126, *p* = 0.725). Most importantly, freezing levels during memory reactivation are notably lower than in this previous study (Rao-Ruiz et al., 2011, Fig. S1D).

**Supplementary Table 1***Power calculations per experiment (achieved power given effect size and sample size, and required sample size for 80% power given effect size).*

| Experiment | FC protocol | Partial η^2^ | # Groups | Total *n* | Achieved power | Required *n* for (1 – β) = .80 |
| --- | --- | --- | --- | --- | --- | --- |
| 1a | 1 × 0.7 mA | 0.001 | 2 | 20 | 0.05 | 7846 |
| 1b^1^ | 1 × 0.7 mA | N/A | 2 | 18 | N/A | N/A |
| 2a | 1 × 1 mA | 0.079 | 2 | 16 | 0.18 | 96 |
| 2b | 1 × 1 mA | 0.136 | 2 | 18 | 0.32 | 54 |
| 3^2^ | 1 × 1 mA | 0.032 | 2 | 18 | 0.11 | 242 |
| 4^1^ | 1 × 1 mA (dark phase) | N/A | 2 | 24 | N/A | N/A |
| 5^1^ | 1 × 1 mA (background CFC) | N/A | 2 | 22 | N/A | N/A |
| 6 | 1 × 1 mA | 0.029 | 3 | 25 | 0.1 | 300 |
| 8 | 3 × 0.5 mA | 0.017 | 3 | 27 | 0.08 | 564 |
| 9a | 3 × 0.3 mA | 0.093 | 3 | 27 | 0.24 | 102 |
| 9b^3^ | 3 × 0.3 mA | 0.008 | 2 | 18 | 0.06 | 978 |

*Note.* ^1^In this experiment freezing reductions from reactivation to testing were stronger in the saline than the propranolol group, hence a power calculation of an amnestic effect of propranolol does not apply here. ^2^ In this experiment the group in which propranolol was dissolved in saline at half the ratio and injected in double the volume/body weight showed smaller – instead – of larger freezing reductions than saline. Therefore, the propranolol double volume group is excluded in this power analysis as well. ^3^ In this experiment the propranolol in normal volume group showed smaller fear reductions than the saline group and was thus also excluded from the power analysis.

**Supplementary Table 2***Tests of increases in freezing from conditioning (before shock delivery) to reactivation, per experiment (paired samples t-test)*

| Experiment | FC protocol | Mean (SD)  Pre-conditioning | Mean (SD)  Reactivation | *t* | df | *p* |
| --- | --- | --- | --- | --- | --- | --- |
| 1a | 1 × 0.7 mA | 3.05 (3.87) | 42.42 (14.64) | 13.336 | 19 | < .001 |
| 1b | 1 × 0.7 mA | 4.96 (2.94) | 52.65 (12.84) | 15.743 | 17 | <.001 |
| 2a | 1 × 1 mA | 2.00 (2.02) | 45.20 (14.66) | 12.329 | 15 | <.001 |
| 2b | 1 × 1 mA | 2.27 (1.80) | 43.53 (13.55) | 13.083 | 17 | <.001 |
| 3 | 1 × 1 mA | 0.50 (0.96) | 29.68 (19.18) | 7.893 | 26 | <.001 |
| 4 | 1 × 1 mA  (dark phase) | 0.39 (0.51) | 14.73 (11.57) | 6.03 | 23 | <.001 |
| 5 | 1 × 1 mA (background CFC) | 0.61 (1.47) | 36.75 (21.10) | 8.048 | 21 | <.001 |
| 6 | 1 × 1 mA | 0.09 (0.32) | 21.24 (14.63) | 6.751 | 22 | <.001 |
| 7 | No shock | 0.11 (0.27) | 0.92 (1.31) | 2.003 | 11 | 0.070 |
| 7 | 1 × 0.7 mA | 0.47 (0.97) | 23.60 (17.40) | 4.627 | 11 | <.001 |
| 7 | 1 × 1 mA | 0.30 (0.38) | 30.56 (17.33) | 6.012 | 11 | <.001 |
| 8 | 3 × 0.5 mA | 0.07 (0.24) | 57.86 (21.33) | 14.108 | 26 | <.001 |
| 9a | 3 × 0.3 mA | 0.16 (0.41) | 23.84 (15.46) | 7.916 | 26 | <.001 |
| 9b | 3 × 0.3 mA | 0.17 (0.44) | 17.68 (15.79) | 5.783 | 26 | <.001 |

**References**

Rao-Ruiz, P., Rotaru, D. C., Van Der Loo, R. J., Mansvelder, H. D., Stiedl, O., Smit, A. B., et al. (2011). Retrieval-specific endocytosis of GluA2-AMPARs underlies adaptive reconsolidation of contextual fear. *Nat. Neurosci.* 14, 1302–1308. doi:10.1038/nn.2907.
